# Identification of an *SRY*-negative 46,XX infertility male with a heterozygous deletion downstream of *SOX3* gene

**DOI:** 10.1186/s13039-022-00580-7

**Published:** 2022-02-14

**Authors:** Shengfang Qin, Xueyan Wang, Jin Wang

**Affiliations:** Department of Medical Genetics and Prenatal Diagnosis, Sichuan Provincial Maternity and Child Health Care Hospital, Chengdu, 610045 Sichuan China

**Keywords:** 46,XX male, *SRY*-negative, Fluorescence in situ hybridization, Chromosome microarray chip, Whole genome analysis, Sex development and differentiation

## Abstract

**Background:**

A male individual with a karyotype of 46,XX is very rare. We explored the genetic aetiology of an infertility male with a kayrotype of 46,XX and *SRY* negative.

**Methods:**

The peripheral blood sample was collected from the patient and subjected to a few genetic testing, including chromosomal karyotyping, azoospermia factor (*AZF*) deletion, short tandem repeat (STR) analysis for *AMELX, AMELY* and *SRY*, fluorescence in situ hybridization (FISH) with specific probes for CSP 18/CSP X/CSP Y/*SRY*, chromosomal microarray analysis (CMA) for genomic copy number variations(CNVs), whole-genome analysis(WGA) for genomic SNV&InDel mutation, and X chromosome inactivation (XCI) analysis.

**Results:**

The patient had a karyotype of 46,XX. *AZF* analysis showed that he missed the *AZF* region (including a, b and c) and *SRY* gene. STR assay revealed he possessed the *AMELX* in the X chromosome, but he had no the *AMELY* and *SRY* in the Y chromosome. FISH analysis with CSP X/CSP Y/*SRY* showed only two X centromeric signals, but none Y chromosome and *SRY*. The above results of the karyotype, FISH and STR analysis did not suggest a Y chromosome chimerism existed in the patient's peripheral blood. The result of the CMA indicated a heterozygous deletion with an approximate size of 867 kb in Xq27.1 (hg19: chrX: 138,612,879–139,480,163 bp), located at 104 kb downstream of *SOX3* gene, including *F9*, *CXorf66*, *MCF2* and *ATP11C*. WGA also displayed the above deletion fragment but did not present known pathogenic or likely pathogenic SNV&InDel mutation responsible for sex determination and development. XCI assay showed that he had about 75% of the X chromosome inactivated.

**Conclusions:**

Although the pathogenicity of 46,XX male patients with *SRY* negative remains unclear, *SOX3* expression of the acquired function may be associated with partial testis differentiation of these patients. Therefore, the CNVs analysis of the *SOX3* gene and its regulatory region should be performed routinely for these patients.

## Background

46,XX sex reversal is a disease caused by the abnormalities of ovarian development, characterized by 46,XX karyotype and male phenotype, often referred to as 46,XX male [1]. 46,XX male is very rare, and the incidence rate in male newborns is about 1/20,000 [2]. 46,XX male is often classified into 46,XX(*SRY*+) male or 46,XX(*SRY*-) male according to the presence or absence of *SRY* gene in his genome. 46,XX(*SRY*-) males are only a minority of 46,XX males, as the percentage of 46,XX(*SRY* +) males caused by *SRY* translocation and Y chromosome chimerism is more than 90% [3–5]. There are some causes of 46,XX(*SRY*-) males previously reported, such as the dose change or mutation of *SOX9* gene [6, 7], loss of function mutation of ovarian stimulating gene *WNT4* or *RSPO1* gene [8, 9], heterozygous mutation of *NR5A1* gene [10, 11]. Furthermore, the arrangement of the *SRY*-box transcription factor 3 (*SOX3*) gene was considered as a cause of this disease [12]. We will proceed with the molecular genetic identification of a 46,XX(*SRY*-) male patient admitted to our clinic. The research results will enrich the theoretical knowledge and guide the clinical treatment of this kind of patient.

## Methods

### Subject

A 31-years-old patient, heigh 166 cm and weigh 52.5 kg, went to our clinic due to primary infertility. Physical examination showed a male appearance, a thin beard, Adam's apple, two broad bean-size of testicles and an average size of the penis. No sperm was found in three routine semen tests. Hormone test results were follow: Testosterone: 1.75 ng/mL(reference value range(RVR): 2.80–8.00), progesterone: 0.11 ng/mL(RVR: 0.20–1.40), prolactin: 208.44uIU/mL(RVR: 86.00–324.00), estradiol: 5.00 pg/mL(RVR:27.10–52.20), luteinizing hormone: 29.32mIU/mL(RVR: 1.70–8.60), follicle-stimulating hormone 37.88mIU/mL(RVR: 1.50–12.40). The patient had no siblings and denied family history. His parents are not consanguineous, had no abnormal phenotype, and refused the laboratory testing and physical examination.

### Specimen preparation and DNA extraction

5 mL of venous blood was collected from patients with heparin sodium and EDTA-Na_2_ anticoagulant tubes, respectively, and ready for use. According to the manufacturer’s protocols, genomic DNA was extracted from EDTA-Na_2_ anticoagulated blood using QIAamp DNA Mini Kit (QIAGEN Company). DNA was qualified when the concentration was more than 30 ng/uL, the OD260/280 value between 1.8 to 2.0 determined by ultraviolet spectrophotometer Nanodrop 1C (Thermo Fisher Scientific).

### Chromosome karyotype analysis

Lymphocytes of heparin sodium anticoagulated blood were cultured, harvested, and prepared for microscope slides before Giemsa staining according to conventional cell culture methods. Zeiss karyotype analysis system (Karl Zeiss, Germany) was adopted for chromosome count and karyotype analysis, the same as previous studies [13].

### Azoospermia factor (*AZF*) detection

Multiplex amplified was performed with *AZF* detection kit (Yaneng corp.), then 2.0% agarose electrophoresis and imaging, according to manufacturer’s instructions.

### Short tandem repetition(STR) analysis

STR sites were performed by multiplex fluorescence quantitative PCR amplification with Devyser compact v3 kit (Devyser AB, Sweden). The amplification condition was 95 ℃ for 15 min; 94 ℃ 30 s, 58 ℃ 1 min 30 s, 72 ℃ 1 min 30 s, 27 cycles;72 ℃ for 30 min. The amplified products were subjected to capillary electrophoresis with AB 3500Dx gene analyzer, and the electrophoresis data were analyzed by GeneMapper software. The fluorescence peaks of *AMELX* in Xp22.2, *AMELY* in Yp11.2 and *SRY* in Yp11.31 were used to evaluate the patient’s sex chromosomes. The experimental method was referred to in the previous report [14].

### Fluorescence in situ hybridization (FISH) analysis

Lymphocytes in EDTA-Na2 anticoagulant blood were isolated by lymphocyte separation solution and hybridized by CSP 18/CSP X/CSP Y probe (Jin Pujia corp.), using the same method as previously reported [15]. Meanwhile, the metaphase cells harvested from lymphocyte culture were co-hybridized with CSP X/CSP Y probe (Jinpuga Company) and *SRY* probe (Abbott Company). The MIX-1 was prepared by *SRY* hybridization buffer and *SRY* probe at a ratio of 9:1, and the MIX-2 was made with CSP hybridization buffer and CSP X/CSP Y centromeric probe at a ratio of 4:1, and then added the MIX-1 and MIX-2 to the metaphase cells loaded on the glass slide. The Glass slide was denaturated at 78 ℃ for 10 min and hybridized at 42 ℃ for more than 16 h. Refer to reagent instruction for the experimental operation. A fluorescence microscope observed the fluorescence signal of hybridization.

### Chromosomal microarray analysis (CMA) analysis

500-1000 ng of patient DNA and the equivalent amount of reference DNA was taken for simultaneous experiments. After digestion, the labelled patient sample was mixed with the reference sample and co-hybridized to SurePrint G3 CGH + SNP (180 K) chip. Agilent DNA Microarray Scanner was used to scan the fluorescence signals after the slides were washed. Agilent Feature Extraction Software extracted the data from the images(.tif) and converted it to log-ratios data. Agilent CytoGenomics software was used to analyze CNV. Agilent Technologies provide the reagents, chips, instruments and analytical software. Refer to the instructions for specific methods. CMA analysis mainly adopts some online databases such as OMIM (https://omim.org/), DGV (http://dgv.tcag.ca/dgv/), Decipher (https://decipher.sanger.ac.uk/), ClinGen (https://wwW.clinicalgenome.org/), ClinVar (https://www.ncbi.nlm.nih.gov/clinvar/).

### Whole genome analysis (WGA) analysis

Illumina HiSeq PE150 high-throughput dual-terminal sequencing was performed after random interruption into tiny fragments of DNA, terminal repair, phosphorylation, a-tail addition, connector and library construction. Quality control was carried out on the raw sequencing data to obtain high-quality clean data; Then, fastp software [16] was used for comparative analysis of clean data and human reference genome sequence, and data such as sequencing depth and coverage of the target region were counted and obtained Bam files. Finally, SNP/InDel was detected and annotated based on Bam files to obtain all mutation information. The Haplotyper tool of Sentieo software [17] was used to detect SNP and InDel mutation, and ANNOVAR software [18] was used to annotate the mutation results accompanied by multiple databases (such as dbSNP, 1000G, ESP6500, HGMD, OMIM). Meanwhile, CNVkit software was used to analyze CNV [19].

### X chromosome inactivation detection

The sample DNA of undigested and digested by HpaII, which methylation-sensitive restriction enzyme, was amplified by androgen receptor (*AR*) gene-specific primers and capillary electrophoresis subsequently. *B2M* was used as the reference gene, and the digestion proceeded overnight in a 37 ℃ water bath. Multiplex PCR amplified the samples before and after enzyme digestion, respectively. As reported in the literature [20], FAM fluorescein was added to the 5 'end of the forward primer [21]. PCR reaction conditions followed: 95 ℃ for 5 min; 28 cycles of 95 ℃ for 45 s, 58 ℃ for 30 s, 72 ℃ for 30 s; 72 ℃ for 7 min. PCR products were subjected to capillary electrophoresis. XCI ratio was calculated according to formula (d1/u1)/(d1/u1 + d2/u2), and XCI bias was confirmed if the XCI ratio > 70% [22, 23].

## Results

### Result of cytogenetic analysis

**Fig. 1:**
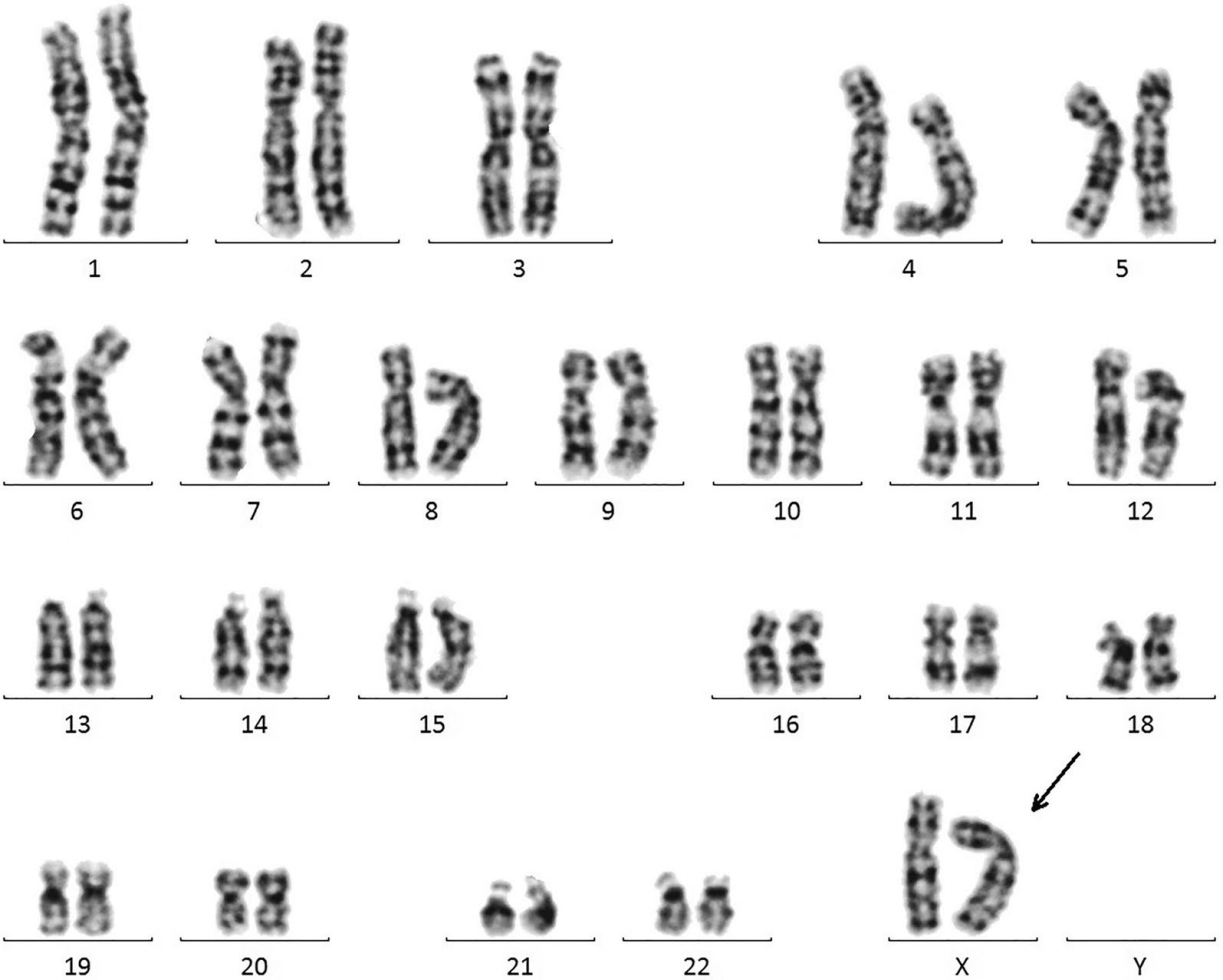
Chromosome karyotype of the patient. The sex chromosomes of the patient are two X chromosomes, as the arrow indicated

The patient's karyotype was 46,XX, as shown in Fig. 1.

### Result of *AZF* detection

The electrophoresis result of the patient showed neither *SRY* nor *AZF* bands (including sY84 and sY86 of *AZF*a, sY127 and sY134 of *AZF*b, sY254 and sY255 of *AZF*c) (The electrophoregram did not show).

### Result of STR analysis

**Fig. 2:**
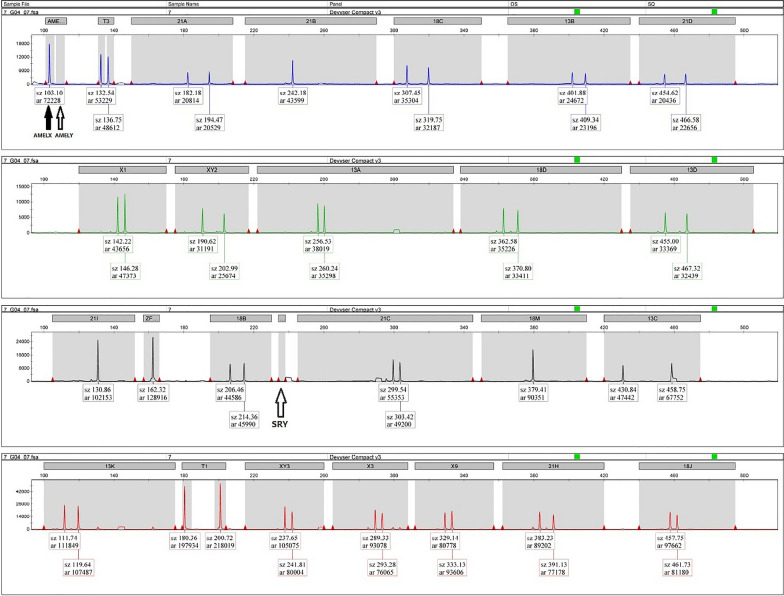
Capillary electrophoresis diagram of STR of the 46,XX (*SRY*-) male patient. The STR result of the patient showed a fluorescence peak of *AMELX* but not that of *AMELY* and *SRY*. The *AMELX*, *AMELY* and *SRY* represent the loci of Xp22.2, Yp11.2 and Yp11.31, respectively. The solid arrow indicated a fluorescence peak of a specific amplicon, while the hollow arrow indicated no

The STR result of the patient showed a fluorescence peak of* AMELX* but not that of the *AMELY* and *SRY*, as shown in Fig. 2.

### Result of FISH analysis

**Fig. 3:**
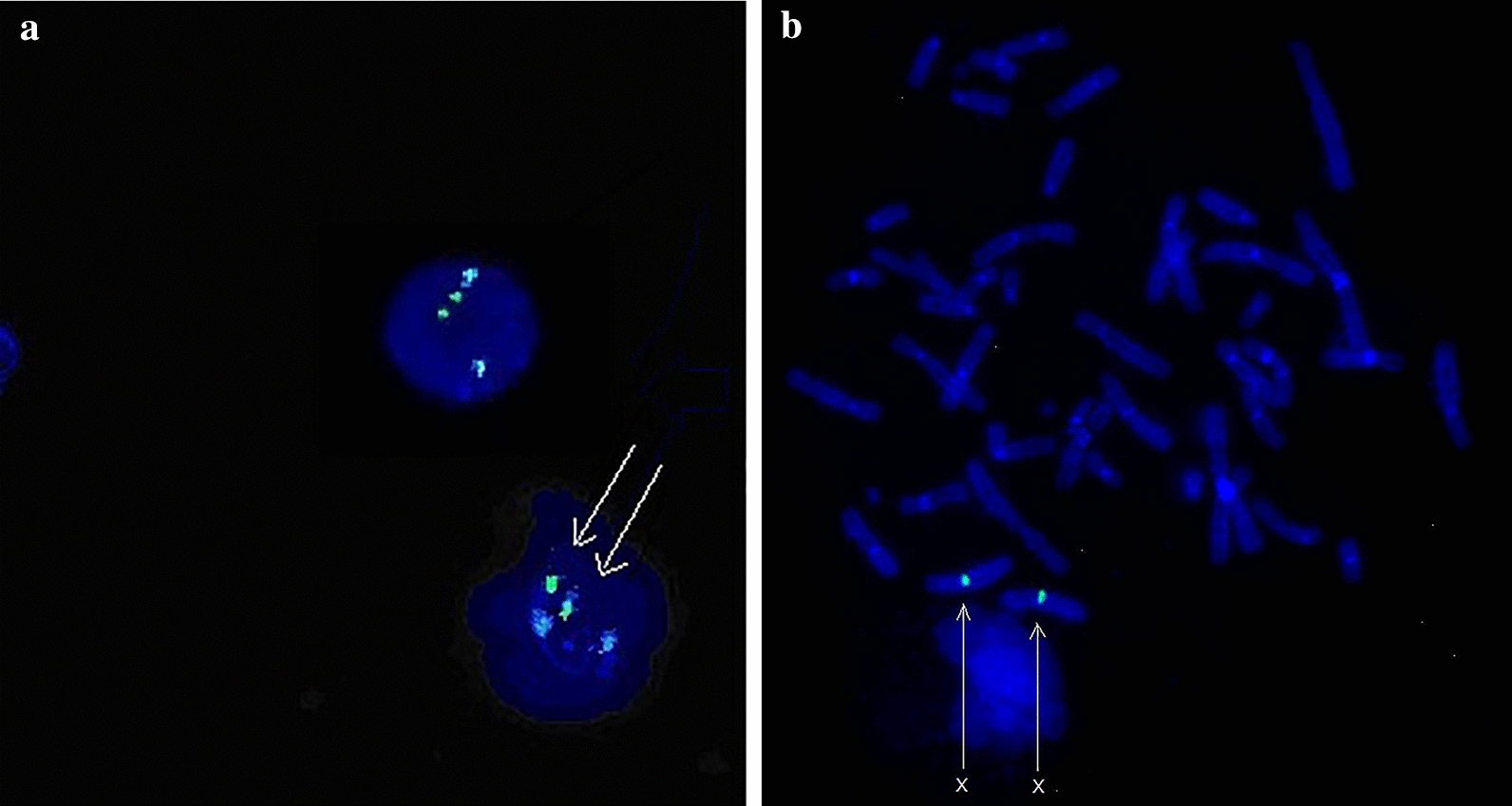
**a** FISH image of interphase cells of the patient detected with the CSP 18, CSP X, and CSP Y probes. The blue signal represented chromosome 18, while the green represented chromosome X, as indicated by the arrows. **b** FISH image of metaphase cells of the patient hybridized with the CSP X, CSP Y and *SRY* probes. The green represented chromosome X, as indicated by the arrows

FISH result of interphase cells hybridized by CSP 18/CSP X/CSP Y probes showed two 18 and two X fluorescence signals but no Y signal, as shown in Fig. 3a. Another FISH result of metaphase cells hybridized with CSP X/CSP Y/*SRY* probes showed two green signals of X chromosome centromere, but not that of *SRY* and Y chromosome, as shown in Fig. 3b.

### Results of Y chromosome chimerism analysis

A single Y chromosome was not found in 200 cells in karyotype analysis. Moreover, Neither *AMELY* and *SRY* fluorescence peaks were detected in the STR analysis results, nor Y chromosome and *SRY* fluorescence signals were shown in the FISH result. A comprehensive evaluation of the above detection results showed no Y chromosome chimerism in the patient's peripheral blood.

### Results of CMA analysis

**Fig. 4:**
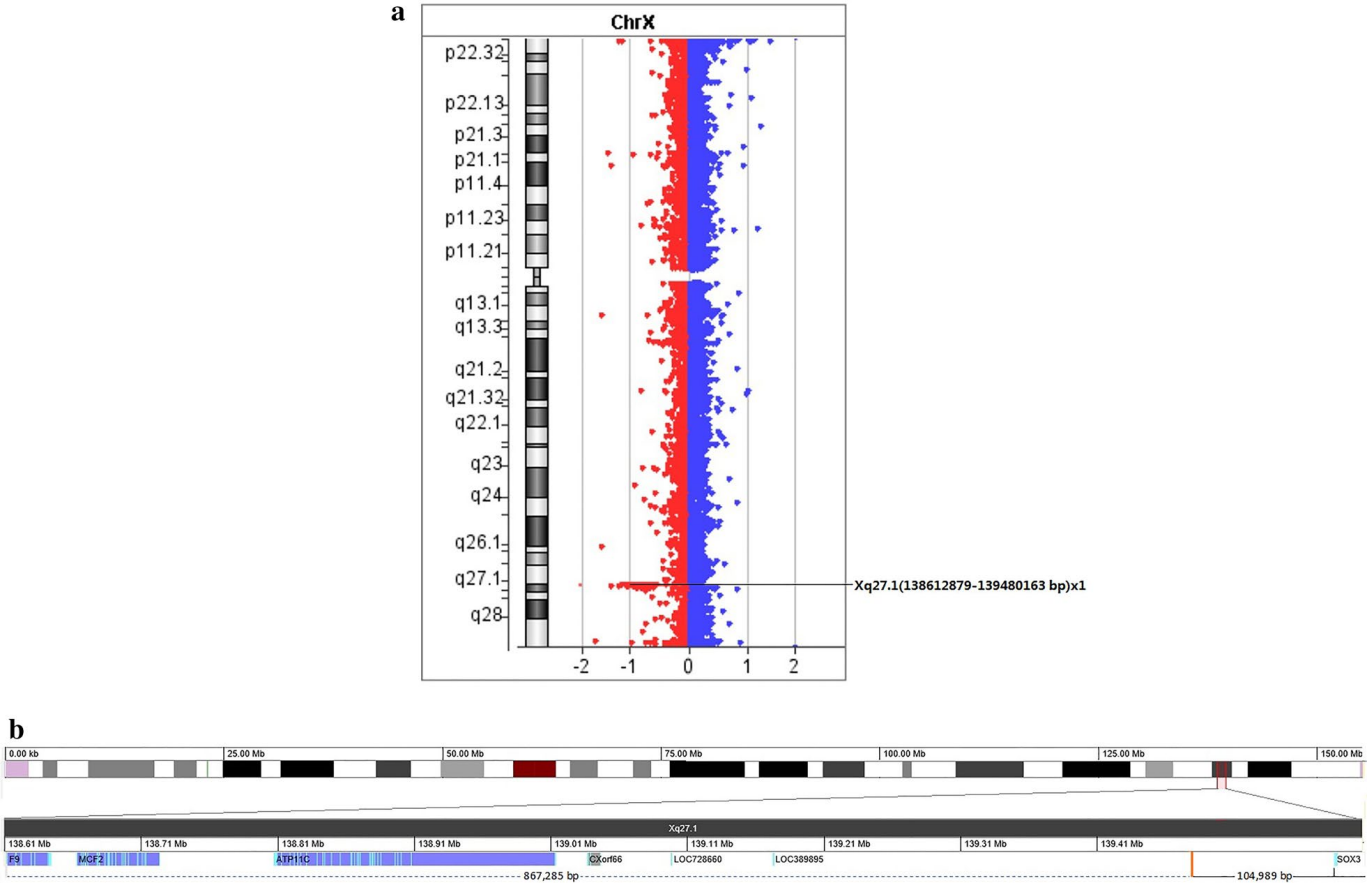
**a** CMA results of the 46,XX (*SRY*-) male patient. There was about 867 kb heterozygous deletion in Xq27.1 (hg19: chrX: 138,612,879–139,480,163 bp), as the line indicated. **b** Schematic diagram of deletion region of the 46,XX (*SRY*-) male patient. The deletion region (hg19: chrX: 138,612,879–139,480,163 bp) is located at 104 kb downstream of the SOX3 gene in Xq27.1. The dotted line indicated the deletion region of 867 kb

Taking 46,XX female genomes as a standard reference, the patient's Xq27.1 (hg19: chrX: 138,612,879–139,480,163 bp) had about 867 Kb of heterozygotic deletion, and the deletion region was located at 104 Kb downstream of the *SOX3* gene, including *F9*, *CXorf66*, *MCF2*, *ATP11C* four protein-coding genes, as shown in Fig. 4a and b.

### Results of WGA analysis

The WGA results showed no pathogenic or likely pathogenic SNV&InDel variant related to sexual development, which can clearly explain the patient phenotype; meanwhile, the results also showed about 892 kb heterozygous deletion (hg19: chrX: 138,609,392–139,501,392) in Xq27.1.

### Results of XCI analysis

**Fig. 5:**
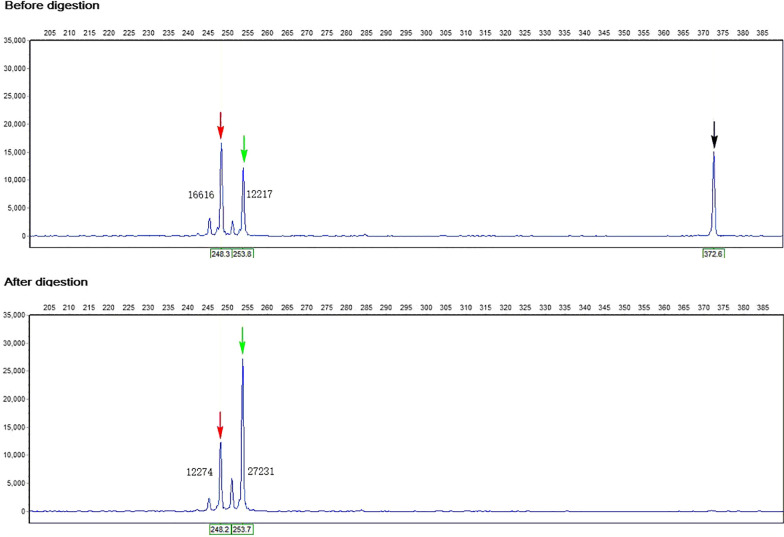
The X chromosome inactivation results of the 46,XX (*SRY*-) male patient. The ordinate and abscissa represent fluorescence intensity and fragment length. The figures near the fluorescence peaks indicate the height of fluorescence peaks. The black arrow indicates the amplified products of the reference gene. After complete digestion, there is no amplified products peak (as shown below). The red and green arrows indicate the two alleles of the AR gene in the X chromosomes. X chromosome inactivation ratio was calculated according to the formula (d1/u1)/(d1/u1 + d2/u2)*100%. This patient's X chromosome inactivation ratio was about 75%, which was non-random. d1: the height of the higher peak after enzyme digestion, u1: the height of the undigested peak, which corresponds to d1; d2: the height of the shorter peak after digestion; u2: the height of the undigested peak, which corresponds to d2

The XCI ratio of the patient was about 75% (as shown in Fig. 5), which indicated he had a non-random inactivation of the X chromosome.

### Comparison of the clinical phenotypes of 46,XX *SRY*-negative male patients with CNV of *SOX3*

Table 1 summarises the clinical features of 46,XX males with *SRY*-negative individuals involved in the CNV of *SOX3*. The patients all had the typical male appearance and showed common abnormal phenotypes, including spermatogenous testicular dysplasia, because they were absent from the entire Y chromosome. Among the seven patients, five patients (case 1, 3, 4, 5, 6) had microduplications spanning the entire *SOX3* gene, another two patients, including case 2 and our patient, had microdeletions near the *SOX3* gene, which were speculated to play a regulatory role for *SOX3* expression. Our patient showed a CNV near the *SOX3* gene in CMA, and WGA ruled out other SNV&InDel mutations associated with sex determination and development. Meanwhile, he had a skewed X chromosome inactivation, which was not inconsistent with case 1.

**Table 1:** Comparison of the clinical phenotypes of 46,XX *SRY*-negative male patients with CNV of *SOX3*

Case	References	CNV	Clinical phenotype	Detection method	X chromosome inactivation
1	Sutton E et al. 2011[12]	The patient contained two microduplications of approximately 123 kb and 85 kb, the former of which spanned the entire *SOX3* gene	The patient was an infertility male of 30-year-old. His height was 165 cm, and he weighed 64 kg, with no abnormal symptoms. Infertility was indicated by two spermograms, which confirmed azoospermia. The patient presented with small testicles and typical secondary sexual characteristics	CMA	X-inactivation studies showed no evidence for skewed inactivation in DNA derived from lymphocytes
2	Sutton E et al. 2011[12]	The patient contained a single 343-kb microdeletion immediately upstream of *SOX3*; the coding sequence of *SOX3* is not affected It is suggested that altered regulation (and not increased dosage) of *SOX3* is the cause of XX male sex reversal	The patient was a 35-year-old with gender dysphoria. Height was 167.5 cm, weight 73.5 kg, with no medical problems apart from ongoing gender identity issues. The external genitalia was typical male, apart from small, soft 6-ml testes. There was little body hair. Primary hypogonadism, with FSH and LH elevated, and testosterone low. Histological examination showed that atrophic changes in the testes, without normal spermatogenesis, thickening and hyalinization of the tubular basal lamina, and diminished interstitial cells. The patient was *SRY*-negative in both peripheral blood and testicular tissue. The *SOX3* rearrangement was not present in his mother	CMA	NA
3	Sutton E et al. 2011[12]	The patient has a large duplication fragment (approximately 6-Mb) encompassing *SOX3* and at least 18 additional distally located genes. The overexpression of *SOX3* probably contributes to the phenotypic complexity. Notably, the proximal breakpoint falls within the *SOX3* regulatory region	The patient was a boy of 19 months who presented some complex phenotypes, including scrotal hypoplasia, microcephaly, unilateral small testis, developmental delay, growth retardation. There were no significant problems during pregnancy or the newborn period. No endocrine evaluation or parental DNA was available	CMA	NA
4	Moalem S et al. 2012[24]	The patient has a de novo copy number gain of 494-kb in the region of Xq27.1 (NCBI 36/hg18: 139,354,859–139,848,664), containing the *SOX3, RP1-177G6.2*, *CDR1* and *MIR320D2* genes	The patient was a 46,XX male newborn with hypospadias. Ultrasound examination revealed normal testicular size and structure	CMA	NA
5	Grinspon RP et al. 2016[25]	The patient has a de novo gain at Xq27.1. The duplicated region was around 0.5 Mb, and encompassed the *SOX3* gene (arr[hg19] Xq27.1(139,541,737–140,043,863) × 3). Other genes included were *RPS17P17*, *CDR1*, *MIR320D2*	The patient was a two years six months boy. He had a trophic phallus 32 mm long and 13 mm wide with ambiguous genitalia and bilateral ovotesticular DSD	CMA	NA
6	Tasic V et al. 2019[27]	The patient has a 550-kb duplication at Xq27 (ChrX: 139,360,520–139,908,320), involving *SOX3*, the non -coding RNA *LINC00632*, *AK054921*, *CDR1* and the miRNA *MIR320D2*	The patient was an 11-year-old boy with right kidney hypoplasia and moderate coronal hypospadias. His testes volume was > 4 mL, and the penis length was 5 cm	CMA	NA
7	Present study	The patient had a heterozygous deletion of about 867 kb in Xq27.1 (138,612,879–139,480,163 bp), located at 104 kb downstream of *SOX3* gene, including *F9*, *CXorf66*, *MCF2* and *ATP11C* in CMA analysis. WGA also displayed the above deletion fragment but did not present known pathogenic or likely pathogenic SNV&InDel mutation responsible for sex determination and development	The patient was a 31-years-old primary infertility patient with two small testicular, his height was 166 cm, and he weighed 52.5 kg. Physical examination showed a male appearance, a trim beard, Adam's apple, the testicles are the size of broad bean, the penis is average size. No sperm was found in routine semen examination. Hormone test results showed that testosterone and estradiol were low and that FSH and LH were high. The parental sample was unavailable	CMA, WGA	The X chromosome inactivation ratio of the patient was about 75%, which was non-random inactivation

## Discussion

The *SRY* gene is recognized as the best TDF candidate gene. As long as the *SRY* gene exists in the individual's genome, male gonadal development will occur even without the Y chromosome; this is the primary mechanism of 46,XX(*SRY* +) male pathogenesis. Therefore, we should pay more attention to the Y chromosome chimerism in 46,XX(*SRY*-) males and rule out as much as possible. The Y chromosome was not found in our patient's peripheral blood derived from the mesoderm through several experimental analyses. However, the possibility of hidden Y chromosome mosaicism in other tissues developed from the endoderm or ectoderm cannot be excluded completely. Of course, it is not easy to find that mosaicism exists in the gonad tissue due to its unavailability.

The rearrangement of the *SOX3* gene has confirmed the causes of some 46,XX(*SRY*-) male individuals in previous reports [12, 24]. *SOX3* gene is one of 20 SOX(*SRY*-related HMG-box) gene family members. Stevanovic et al. cloned the *SOX3* gene and identified its location at Xq27.1 in 1993 [25]. Its sequence is highly conserved among different species. The *SOX3* gene is ancestral to the *SRY* gene [12, 26], so it has high homology with *SRY* and other SOX family genes. *SOX3* gene consists of a single exon and contains an HMG box, encoding 446 amino acids of a transcription factor sox3 protein expressed in vertebrate embryos' central nervous system [27]; it plays a vital role in the pituitary, craniofacial and adrenal development. The variation of the *SOX3* gene is associated with X-linked mental retardation, growth hormone deficiency, X-linked hypothyroidism, 46,XX male sex reversal, and other diseases [12, 28–31].

Loss-of-function of *SOX3* gene mutation did not cause the abnormality of sex determination in mice and humans [32]. However, studies in transgenic mice had shown that in-situ expression of *SOX3* in bipotent gonads resulted in up-regulation of Sox9 expression, testicular induction and XX male sex reversal. Moalem S et al. provided evidence of the duplication of the *SOX3* in XX bipotent gonads causing the acquisition of the *SOX3* function [33], which was related to partial testis differentiation in XX mice lacking the *SRY* gene. Moreover, overexpression of *SOX3*, synergistically expression with *SF1*, up-regulated *SOX9* stimulated gonad development into testis in XX mice [12]. The *SRY* gene was derived from regulatory region mutation of the *SOX3* gene and expressed in the early gonad. The data of transgenic mice indicated that *SOX3* and *SRY* were interchangeable in sex determination function.

The variation types of the *SOX3* gene associated with 46,XX males were all the CNV in previous reports. To date, 6 cases of 46,XX males related to the *SOX3* CNV have been reported. In some cases, the copy number duplication of the *SOX3* gene resulted in changing gene product dose [33–35]; among other cases, it was speculated that the CNV had a position effect on the *SOX3* gene expression because the CNV did not contain the *SOX3* gene but was only close to the region of the *SOX3* gene, or the breakpoint of CNV fell in the regulatory region of *SOX3 *[12, 36]. CMA showed that our patient had a heterozygous deletion of about 867 kb in Xq27.1 (hg19: chrX: 138,612,879–139,480,163 bp), which was located at 104 kb downstream of the *SOX3* gene, including *F9*, *CXorf66*, *MCF2* and *ATP11C*; Meanwhile, whole-genome sequencing also found an 892 kb heterozygosity deletion in Xq27.1 (hg19: chrX: 138,609,392–139,501,392), and no SNV&InDel mutation associated with abnormal sex determination and development. No similar report was found in the DGV database about this deletion area, and no sexual reversal phenotype was reported in Decipher and ClinVar databases. Similar to previous reports [12, 34], we speculated that the deletion region might involve the regulation region of the *SOX3* gene, leading to determination and differentiation of male testis through weakening inhibition of *SOX3* and increasing expression *SOX3 *[12, 24, 35].

Similar to the two adults patients reported by Sutton E et al. [12], the main features of our patient are azoospermia and infertility [37] because of a deletion of the entire Y chromosome. Microspermatocentesis is not recommended for this patient because his testis produces no sperm, and the plan of donated sperm was introduced to him.

Since the deletion of our patient existed in the X chromosome, the XCI factor affecting the clinical significance should be considered. The XCI is a dose-compensation mechanism in XX individuals. It usually occurs in early embryonic development as one of the X chromosomes in a woman is inactivated, with only one paternal or maternal chromosome being expressed in each cell of the female individual. In general, XCI is random, i.e., the inactivation ratio of the two X chromosomes in females is 50%: 50% [38]. In 46,XX (*SRY*+) males, some studies [21, 39] showed a high degree of XCI bias (greater than 90%). Their phenotype differed with the variable inactivation of the X chromosome carrying the *SRY* gene [40, 41]. Here, we conducted the XCI analysis of the peripheral blood in 46,XX male(*SRY*-) patient for the first time. The XCI ratio was about 75%, a moderate XCI bias. We speculate that the expression of positive selection of the deficient X chromosomes, which contained the deleted fragment, results in the development of male gonads of the patient, similar to previous reports [42]. The XCI ratio of gonads tissues may differ from peripheral blood [22]. However, it cannot be accurately known because the sample is inaccessible. We could not know the origin of the Xq27.1 microdeletion in the downstream region of the *SOX3* gene of the patient because the relevant results of the patient's parents were not available. We have a tentative assumption that the CNV might regulate the *SOX3* gene expression, thus leading to the patient with a karyotype of 46,XX *SRY*-negative developing into a male. The pathogenicity of CNV has not been confirmed, and this is our upcoming research task to be performed.

At present, the pathogenesis of *SRY* positive 46,XX male patients is relatively straightforward. However, for *SRY* negative 46,XX male, the molecular mechanism, signalling pathway, and genetic regulation are remain unknown, and the diagnosis and treatment are still relatively complex. Sutton et al. [12] identified three arrangements containing or adjacent to the *SOX3* gene, accounting for 19% (3/16) in 16 *SRY*-negative 46,XX male patients. Subsequently, several case reports indicated that *SOX3* was a critical pathogenic factor of these patients. Therefore, it is crucial to conduct the CNV determination of the *SOX3* gene in all 46,XX(*SRY*-) males. It is noteworthy that the current and reported *SOX3* duplications or deletions are below the detection threshold of conventional karyotype and can be found using CMA. Therefore, CMA analysis is routinely recommended to the CNV of *SOX3*, and high-throughput sequencing such as WGA can simultaneously proceed to exclude other SNV/INDEL mutations. In addition, the XCI analysis of these patients also should be considered.

## Data Availability

The datasets used and analysed during the current study are available from the corresponding author on reasonable request.
